# Relationships Between Personality Features and the Rubber Hand Illusion: An Exploratory Study

**DOI:** 10.3389/fpsyg.2019.02762

**Published:** 2019-12-10

**Authors:** Dalila Burin, Claudia Pignolo, Francesca Ales, Luciano Giromini, Maria Pyasik, Davide Ghirardello, Alessandro Zennaro, Miriana Angilletta, Laura Castellino, Lorenzo Pia

**Affiliations:** ^1^Kawashima Laboratory, Institute of Development, Aging and Cancer and Smart-Aging Research Center, Tohoku University, Sendai, Japan; ^2^Department of Psychology, University of Turin, Turin, Italy; ^3^SpAtial, Motor and Bodily Awareness Research Group, Department of Psychology, University of Turin, Turin, Italy; ^4^Neuroscience Institute of Turin, Turin, Italy

**Keywords:** rubber hand illusion, body ownership, personality traits, Rorschach, Personality Assessment Inventory

## Abstract

The rubber hand illusion paradigm allows investigating human body ownership by inducing an illusion of owning a life-sized fake hand. Despite the wide consensus on the fact that integration of multisensory signals is the main interpretative framework of the rubber hand illusion, increasing amount of data show that additional factors might contribute to the emergence of the illusion and, in turn, explain the strong inter-individual differences of the illusory patterns. Here, we explored whether and how personality features contribute to the emergence of the illusion by administering to healthy participants the rubber hand illusion paradigm along with two well-known personality tests, i.e., the Personality Assessment Inventory (PAI) and the Rorschach test. Results showed that two Rorschach domains (i.e., “Perception and Thinking Problems” and “Self and Other Representation”) were positively correlated with the illusory mislocalization of the own left hand toward the fake hand. Further analyses suggested that while the tendency to perceive unconventionally is related to mislocalizing the own hand toward the fake hand, the association of the RHI index and other personality features measured by the Rorschach remain uncertain. However, our findings in general suggest that personality features might have a role in the emergence of the rubber hand illusion. This, in turn, could explain the high inter-individual variability of the illusory effects.

## Introduction

In psychological sciences, there is a wide consensus on the fact that human’s sense of self is strongly rooted in bodily related processes (Gallese and Sinigaglia, 2010). One of the key components of such complex construct is body ownership, i.e., the conscious experience of the body as one’s own (Gallagher, 2000). This omnipresent and ubiquitous feeling is crucial for survival since it allows interacting with the environment and experiencing the boundaries between our own body and the external world.

Research in cognitive neuroscience has shown that a key component of body ownership is the integration of several incoming signals (e.g., visceral, interoceptive, proprioceptive, visual, tactile), which are mapped in the brain at levels of growing complexity (e.g., Damaasio, 2010). For instance, when a butterfly lands on our own arm, visual, tactile, and proprioceptive signals are matched in spatiotemporal terms. This triggers the feeling that the unique source of these sensations is our own physical body. Such interpretation has been built up mainly through an experimental manipulation that alters this experience by creating a mismatch among some of these signals. Such paradigm, known as the Rubber Hand Illusion (hereinafter RHI), shows that temporally synchronous (but not asynchronous) touches onto a visible fake hand and onto the hidden participant’s hand produces the compelling feeling of ownership of the fake hand (Botvinick and Cohen, 1998; Farnè et al., 2000; Ehrsson et al., 2004; Tsakiris and Haggard, 2005; Costantini and Haggard, 2007; Longo et al., 2008; Kammers et al., 2009; Tsakiris et al., 2011; Kalckert and Ehrsson, 2014; Burin et al., 2017a, 2018). The effect is thought to arise because visual information has more weight in influencing the final percept. Specifically, the initial conflict between tactile/proprioceptive sensation of the own hand and vision of the rubber hand is resolved by integrating those signals in a coherent, but impossible percept: a fake hand sensed as part of one’s own body. The illusion is quantified by means of both explicit and implicit measures that can capture different, albeit partially related, components of the illusory experience (Rohde et al., 2011). The implicit measure, known as proprioceptive drift, represents the perceptual mislocalization of the own hand toward the rubber hand (i.e., the spatial difference between the felt position of their own hand before and after experimental manipulation). On the contrary, the explicit measures refer to the subjective experience of owning the fake hand, which is measured by using an *ad hoc*, self-report questionnaire. To what extent these measures are related to different or same central process is still debated (Abdulkarim and Ehrsson, 2016). Importantly, the mere spatiotemporal correlation between tactile and visual stimuli is a necessary but not sufficient condition to trigger the illusion. Indeed, some additional constraints imposed by a preexisting internal representation of the body should be satisfied. Specifically, the illusory effect does not occur with a neutral object, with the fake hand in incongruent positions (e.g., in a third person perspective), or with different stroking directions (Ehrsson et al., 2004; Tsakiris and Haggard, 2005; Costantini and Haggard, 2007; Limanowski et al., 2014; Burin et al., 2017b). Hence, the RHI effects require both bottom–up and top–down signals. Specifically, whenever the delivered stimuli are temporally and spatially synchronous (i.e., bottom–up signals), and whenever the fake hand is congruent with the preexisting representation of the body in terms of postures and identity (i.e., top–down signals), the conflict between somatosensory (i.e., touch and proprioception) representation of the own hand and vision of a fake hand is resolved by the incorporation of the fake hand within the participant’s own body (Costantini and Haggard, 2007; Makin et al., 2008; Tsakiris, 2010).

Despite the strong agreement on the above-mentioned interpretation based on two different categories of signals (i.e., multisensory integration and preexisting body representations), increasing evidence suggests that they might not be sufficient conditions to induce the illusion. Firstly, there is an extreme inter-individual variability in terms of RHI susceptibility within human population (Haans et al., 2012), with people reporting the illusion by simply seeing the fake hand (Rohde et al., 2011), others claiming its changing appearance (Lewis and Lloyd, 2010), others only having an impression of strangeness, and others experiencing no illusory experience at all (Wold et al., 2014). Secondly, the illusion strength is modulated by a variety of different additional factors other than the ones mentioned above: attentional fluctuations (Haans et al., 2012), interoceptive sensitivity (Tsakiris et al., 2011), temporal resolution in multisensory perception (Costantini et al., 2016), degree of empathic concern (Durgin et al., 2007; Asai et al., 2011), psychosis proneness (Asai et al., 2011; Thakkar et al., 2011; Germine et al., 2013; Kallai et al., 2015), malleability of body image (Eshkevari et al., 2012), emotional intelligence (Perepelkina et al., 2017) and hypnotic (Walsh et al., 2015)/sensory (Marotta et al., 2016) suggestibility.

In line with the above-mentioned considerations, we conducted an exploratory study aimed at examining whether specific personality and/or psychopathological features might contribute to explain the high inter-individual variability of the RHI. Consistently with the well-known high intra-individual long-term stability of the illusory effects (Bekrater-Bodmann et al., 2012), we hypothesized that persistent personality characteristics, in addition to temporary states, might be related to some degree to the susceptibility to the illusion. Furthermore, we wanted to shed light on the possible correlation between explicitly stated personality/psychopathology features (i.e., self-reported) and explicit indexes of the RHI, and between implicitly assessed personality/psychopathology characteristics (i.e., those that are performed rather than verbally reported) and implicit indexes of the RHI effects). Last but not least, since some studies showed a difference in the illusion strength for the two hands (e.g., Ocklenburg et al., 2011; Bertamini and O’Sullivan, 2014; Marotta et al., 2016; Dempsey-Jones and Kritikos, 2019), we explored correlations separately for the two hands.

To that goal, we administered the RHI paradigm and two widely known measures of personality and psychopathology to a sample of healthy participants. Specifically, the self-reported personality/psychopathology features were assessed by using the Personality Assessment Inventory (hereinafter, PAI; Morey, 1991, 2007), whilst the more implicit, performance-based personality and psychopathological characteristics were assessed with the Rorschach test (Rorschach, 1921) in its most recent and updated version, that is the Rorschach Performance Assessment System (R-PAS; Meyer et al., 2011). Then, we examined the correlations between the implicit and the explicit measures of RHI and the personality/psychopathology features assessed by the above-cited tools.

As for the PAI, the test was developed to measure both the diversity of elements subsumed within a specific construct and the severity of different symptom manifestations. For example, the Depression (DEP) scale with its three subscales covers worthlessness and hopelessness (Cognitive, DEP-C), feelings of sadness and anhedonia (Affective, DEP-A), and problems in the physical functioning (Physiological, DEP-P), so that the more compromised these aspects are, the higher the DEP scale score will be. In addition, the transformation from raw to T scores allows comparing the examinee’s scale scores with the normative, reference sample. For example, a score of 50T would suggest that the examinee experiences depressive features as the majority of community people do (e.g., feeling sadness only sometimes). Scores approaching 70T (i.e., two standard deviations above the mean) may suggest that the examinee is feeling sad, worthless, indecisive most of the time, with changing in the sleep and diet patterns. Given its ability to provide information on both the healthy and pathological ends of most psychological and psychopathological features, we selected the PAI to evaluate self-reported personality features from the examinee’s point of view (Cronbach, 1949; Meyer, 2018).

On the other hand, being the most researched test dealing with typical performances – that is when the individual is left to own predilections (Cronbach, 1949; Teglasi et al., 2012; Meyer, 2017), the Rorschach test allows to observe the personality in action (Meyer et al., 2011). Said differently, asking the examinee to look at the inkblots and to answer to the question “what might this be?” sets in motion cognitive and emotional processes leading the examinee to solve the task (Meyer, 2017). Indeed, completing this standardized task involves looking at the inkblot, giving a visual attribution to the stimulus, providing verbal and non-verbal communications about what the examinee has seen, and interacting with the examiner and the inkblots. The Rorschach test provides an *in vivo* demonstration of personal characteristics that may reside outside consciousness. Indeed, despite the relatively strong correlations found when inspecting external or behavioral criteria, the mean validity of the Rorschach variables was *r* = 0.08 when self-report measures are taken into account (Mihura et al., 2013) suggesting that the Rorschach may provide additional data on the psychological and personality functioning of the examinee.

## Materials and Methods

### Participants

Ninety-six right-handed (Oldfield, 1971) healthy undergraduate student participants with no previous neurological/psychiatric disease gave their written informed consent to participate in the study approved by the Bioethical Committee of University of Turin. The majority of the participants were psychology students, females (80.2%), with a mean age of 21.8 years (*SD* = 3.16 years). Participants were naïve to the purposes of the research.

### Rubber Hand Illusion

The paradigm included four different conditions counterbalanced between participants: Left hand synchronous stimulation, left hand asynchronous stimulation, right hand synchronous stimulation, right hand asynchronous stimulation.

A wooden box (60 cm × 40 cm × 20 cm) divided by a perpendicular panel in two equal parts (30 cm × 40 cm × 20 cm) was placed on a table 15 cm in front of the participant. One of the two parts was hidden from the view, whereas the other was open (a 30 cm × 40 cm automotive panel was used to cover this part when necessary). Two square holes (12 cm × 12 cm) on both horizontal sides of the box allowed placing the rubber and the participant’s hand (left or right). After a familiarization with setting and procedures, the rubber hand (left or right) was placed in the open part of the box and aligned with the correspondent participants’ shoulder (left or right). The participant’s arm (left or right) was placed farthest from the body midline in a fixed location of the closed part with fingers and palm facing down. Therefore, the distance between the real and the rubber hand was approximately 25 cm.

Firstly, the experimenter placed the removable panel on the open part of the box (in order to cover also the rubber hand) and put a wooden stick with a previously applied tailor-ruler (0–100 cm) on top of it. The participant was asked to verbally report the number correspondent to the position of his/her index finger (six trials) which was referred to as the pre-proprioceptive judgment (average of six trials). The position of the stick was randomly varied to avoid employing numbers on the ruler as cues.

**FIGURE 1:**
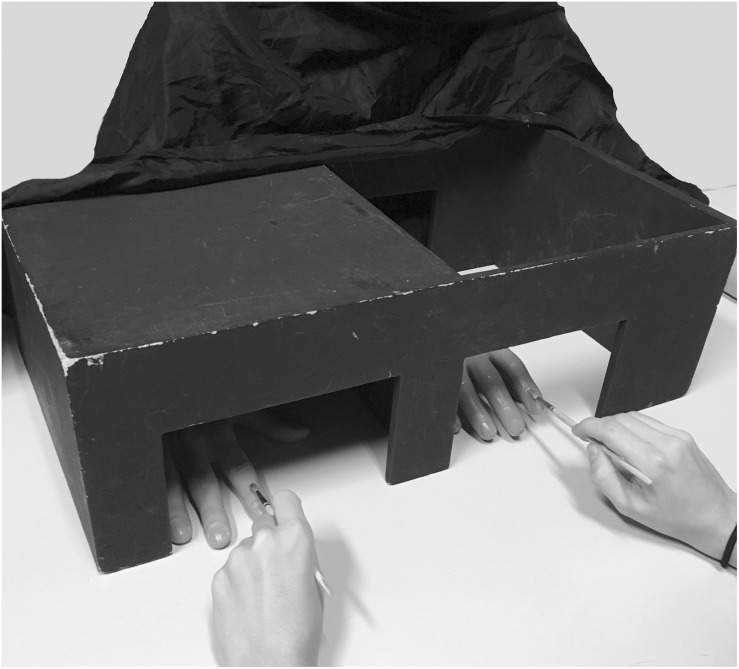
Graphical representation of the RHI set up.

Secondly, the experimenter removed the panel and reminded the participants to always look at the fake index finger. Then, the experimenter started to stroke both the rubber hand’s index finger and the participants’ index finger with two similar small brushes. In both kind of stimulations, the strokes went from the knuckle to the fingertip and this lasted for about 2 s (with a delay of around 500 ms between one stroke and the other). In the synchronous condition, the two index fingers were stimulated simultaneously, whereas in the asynchronous conditions, the fingers were stroked with a random delay of 500 – 1,000 ms. Each block lasted 120 s (see Figure 1 for a graphical representation of the RHI setup).

Thirdly, at the end of each kind of stimulation, the experimenter covered the rubber hand with the panel and, again, asked participants to indicate the position of his/her index finger on the ruler (six trials), which was referred to as the post-proprioceptive judgment (average of six trials). Additionally, participants were also asked to fill out a questionnaire about the experience of the illusion. The questionnaire was composed of six questions (see Supplementary Data Sheet 1) selected from the original RHI questionnaire (Botvinick and Cohen, 1998). Three (Q1 – Q3) aimed to capture different aspects of the illusory perception (e.g., the sensation of touches on the rubber hand and the change in the beliefs of ownership of that hand), whereas three (Q4 – Q6) served as control questions for task compliance and susceptibility effects. The statements were administered in randomized order. Participants rated their agreement/disagreement on a seven-point Likert scale with a range from “+3” (agree very strongly) to “−3” (disagree very strongly) where “0” corresponded to neither agreeing nor disagreeing.

### Personality Assessment Inventory (PAI)

The PAI (Morey, 1991, 2007; Zennaro et al., 2015), a well-known self-report personality and psychopathology assessment tool, can provide a large variety of clinically relevant information. Being a broadband measure, the PAI allows exploring both clinical syndromes (e.g., anxiety, depression) and relevant pathological personality characteristics (e.g., borderline personality features). The PAI is second only to the Minnesota Multiphasic Personality Inventory-2 (MMPI-2; Butcher et al., 1989) in terms of popularity, having surpassed the Millon and the NEO-PI inventories (Mihura et al., 2017); however, compared to the MMPI-2, the PAI presents qualities that make it preferable in terms of psychometric features. First, the PAI consists of 344 items (*vs.* 567 items of the MMPI-2) organized in 22 non-overlapping scales, so that items are scored only on one scale. The PAI scales are divided into 4 validity scales to evaluate response styles, 11 clinical scales to assess the presence of psychopathological syndromes and/or maladaptive personality features, 5 treatment scales to provide information about potential complications in treatment, and 2 interpersonal scales. Moreover, nine of the clinical scales and one treatment scale comprise three to four subscales to further evaluate all the features of the construct being investigated. Also, differently from most of the personality measures, four anchor points were included on the response scale, i.e., (a) “False, not at all true”; (b) “Slightly true”; (c) “Mainly true”; (d) “Very true.” Finally, a recent study has shown that the PAI scores can be organized effectively to make inferences about the five-factor model of personality psychopathology of the DSM-5 (Hopwood et al., 2013).

The PAI was administered individually and participants were required to answer each question without any time limits. Raw scores were computed for the scales, subscales, and supplementary indexes. Subsequently, in accordance with the PAI manual procedure, raw scores were converted into T scores using the Italian normative data, so that a score of 50T represents the mean and a core of 10T represents the standard deviation of the Italian normative sample.

### Rorschach Performance Assessment System

The Rorschach (Rorschach, 1921) is one of the most utilized performance-based personality assessment tools (Camara et al., 2000; Mihura et al., 2017). For this study, we adopted the R-PAS (Meyer et al., 2011), the most updated, valid (Mihura et al., 2013; Su et al., 2015; Giromini et al., 2016), and reliable (Viglione et al., 2012; Pignolo et al., 2017) method to administer, code, and interpret the Rorschach. Indeed, R-PAS is grounded on the most extensive meta-analysis published to date for a personality test (Mihura et al., 2013; Mihura and Meyer, 2018) and it has introduced some important technical modifications compared to previous systems (Meyer and Eblin, 2012). Differently from the past, the Rorschach is now considered a valid and reliable instrument to evaluate personality and psychological features. This is particularly true for R-PAS that values the psychometric soundness of its variables. It is noteworthy that the Society for Personality Assessment (2005) stated “the Rorschach possesses documented reliability and validity similar to other generally accepted test instruments used in the assessment of personality and psychopathology and that its responsible use in personality assessment is appropriate and justified” (p. 221). While it is true that Garb (1999) “called for a moratorium on the use of the Rorschach test in clinical and forensic settings,” the same author recently stated “in light of the compelling evidence laid out by Mihura et al. (2013), the time has come to withdraw this recommendation so far as it applies to the Cognitive Quartet of Rorschach scores. We are convinced that these scores provide valid information regarding cognitive ability and cognitive impairment that can be helpful in some applied and research settings” (Wood et al., 2015; p. 243). Thus, even the harsher Rorschach critics have admitted its validity, lifting their previous claim for a moratorium for those variables that passed the severe exam of the meta-analyses (Mihura et al., 2015; Wood et al., 2015). In particular, the Rorschach variables assessing psychosis proneness and thought disorders received the strongest support (Kleiger, 2017; Mihura and Meyer, 2018).

The test consists of ten inkblot designs, handed to the participant one at a time and asked “What might this be?”. The strategies and approaches the examinee uses to identify, select, and describe his/her responses are operationalized and coded to obtain useful information about his or her personality. According to Meyer et al. (2011), “the best or most valid interpretations are those in which the coded behaviors observed in the microcosm of the task generalize to parallel mental, verbal, and perceptual behaviors in the external environment” (p. 1). Thus, for example, a person who repeatedly sees anatomical contents and/or medical images in the ambiguous inkblots is likely to present bodily concerns or preoccupations in his/her real life too.

R-PAS provides guidelines for standardized administration and coding, and normative reference data for the interpretation of the results. To score the Rorschach, the examiner applies codes to each response about where the response is seen (location), whether the white space is used (space integration, space reversal), what is seen (content), object qualities (synthesis, vagueness, pair), how well the object fits the blot (form quality), whether the object seen by the examinee is the most frequently perceived one in the card (popular), what makes it look like that (determinants), and whether issues with thought processes (cognitive codes) or specific themes (thematic codes) are present (Meyer et al., 2014). Subsequently, the codes are aggregated across all responses of a given protocol by using the R-PAS online scoring program. The scoring program converts raw scores into Standard Scores based on the international, normative reference data, so that a score of 100SS reflects the mean and a score of 15SS reflects the standard deviation of the normative sample.

The R-PAS interpretative output is organized into five interpretative domains: (a) “Administration behaviors and observations”, to evaluate basic task-relevant behaviors observed during the administration; (b) “Engagement and cognitive processing”, to assess the examinee’s complexity, psychological resources, and coping style; (c) “Perception and thinking problems”, to determine the presence of perceptual distortions and thought disorder; (d) “Stress and distress”, to examine the presence of affective discomfort or distress; (e) “Self and other representation”, to understand the examinee’s self-image and interpersonal competency. The R-PAS variables are organized into two main sections: Page 1 includes 35 variables with good support in empirical literature, and Page 2 includes 25 variables with less support in empirical literature, which have however demonstrated enough validity to be included in R-PAS. At a protocol-level of interpretation, Rorschach scores are grounded on clinical observations that have replicated data supporting their construct validity and on behavioral similarity, in that the behavior coded with the Rorschach parallels the behavior acted in every-day life.

### Procedure

To avoid any possible order effects and due to the relatively unpredictable duration of the Rorschach^1^, participants were administered two sessions 1 week apart. Half of the participants were given the Rorschach and the RHI in the first session, and the PAI in the second. The other half were given the opposite order. The order of the Rorschach test and RHI was counterbalanced between participants.

The personality tests and the RHI procedure were administered by two different groups of researchers, so that the researchers who administered the personality tests were blind about the participants’ performance during the RHI procedure (and vice versa). Moreover, both the personality tests and the RHI data were scored and analyzed only after the data collection ended (see section Author Contributions).

### Statistical Analysis

#### Rubber Hand Illusion

As explicit and implicit measures of the RHI effects, we calculated proprioceptive shift and subjective shift. These to indices have been previously employed in the literature (e.g., Grynberg and Pollatos, 2015; Abdulkarim and Ehrsson, 2016; Burin et al., 2017a) and allow obtaining the pure effect of synchronous stimulation.

With respect to proprioceptive shift, pre-proprioceptive judgment was subtracted from post-proprioceptive judgment in each stimulation condition and referred to as proprioceptive drift (Tsakiris and Haggard, 2005). Then, proprioceptive drift in the asynchronous condition was subtracted from proprioceptive drift in synchronous condition and represented proprioceptive shift. Positive values indicated a higher mislocalization toward the rubber hand in the synchronous with respect to the asynchronous condition. For the subjective shift, the averaged ratings of the three control questions (4–6) were subtracted from averaged ratings of the three experimental statements (1–3) in each stimulation condition (Kalckert and Ehrsson, 2012). Then, the ratio between real and control questions in the asynchronous condition was subtracted from the ratio between real and control questions in the synchronous condition and represented the subjective shift. Positive values indicated a higher illusion strength in the synchronous with respect to the asynchronous condition. Before any analysis, we evaluated the normality of the variables’ distribution and, for those departing substantially from normality (i.e., skewness > 2 and kurtosis > 7; West et al., 1995) we computed non-parametric statistics. For both proprioceptive and subjective shift, we ran a dependent samples *t*-test with hand (left or right) as within subjects’ factor.

#### Personality Assessment Inventory

Raw scores were converted into *T*-scores according to the Italian transformation tables (Zennaro et al., 2015), so that scores equal or above 70T suggest clinical problems in that particular area. All variables were normally distributed (West et al., 1995). Then, we evaluated the validity of the PAI profiles by inspecting the Inconsistency (ICN < 73T) and Infrequency (INF < 75T) scales (Morey, 2007), two measures of random, inconsistent responding. All the PAI profiles were valid.

#### Rorschach Performance Assessment System

One Rorschach variable (i.e., Texture; T) did not produce a normal distribution and, thus, we computed non-parametric statistics (i.e., Spearman Rho) for that variable when examining its relationship to RHI. For each of the four R-PAS domains that are found in both Page 1 and Page 2 (i.e., “Engagement and cognitive processing”; “Perception and thinking problems”; “Stress and distress”; “Self and other representation”), a summary score was calculated by computing the mean of the subcomponents’ Standard Scores included in each domain. Given that the subcomponents of a certain domain measure both healthy and pathological features of that clinical construct, in computing the summary scores, some subcomponents were reversed so that the interpretation of all the variables was in the same pathological direction.

#### Correlations Between Rubber Hand Illusion and Personality Features

To investigate the relationship of RHI to personality, we computed Pearson’s correlations between proprioceptive shift and subjective shift to PAI clinical scales and R-PAS domain scores. When a PAI scale score or R-PAS domain score produced a statistically significant correlation, its individual variables, or subcomponents, i.e., the PAI subscales or R-PAS individual variables composing that specific summary score or domain, were investigated too. Because we tested a number of independent statistical tests, we applied the False Discovery Rate (FDR) controlling procedure (Benjamini and Hochberg, 1995).

## Results

### Rubber Hand Illusion

Table 1 shows the descriptive statistics. With respect to the proprioceptive drift, mean values for the synchronous stimulation were positive and higher than asynchronous one for both the left and the right hand confirming the presence of the illusion. Consequently, we employed the proprioceptive shift in the subsequent analysis. The dependent samples t-test comparing the proprioceptive shift by the two hands resulted to be significant (*p* = 0.044) with a significant higher shift for the left than for the right hand. As for the subjective component, values were positive exclusively for real questions in the synchronous condition for both the left and the right hand, so here as well we employed subjective shift. The dependent samples t-test comparing the subjective shift by the two hands was not significant (*p* = 0.713).

**TABLE 1:** Minimum and maximum value, mean and standard deviation (SD), Skew and Kurtosis of RHI, PAI, and Rorschach data.

	**Minimum**	**Maximum**	**Mean**	***SD***	**Skew**	**Kurtosis**
**RHI**						
Proprioceptive Drift Sync Left	–2.3	15.3	1.95	2.77	2.39	8.24
Proprioceptive Drift Async Left	–4	4.5	0.01	1.61	0.18	0.41
Proprioceptive Drift Sync Right	–2.3	9.5	1.37	2.22	1.29	2.44
Proprioceptive Drift Async Right	–4	3.5	0.11	1.53	0.05	0.15
Proprioceptive Shift Left	–3.5	16.5	1.94	3.17	1.53	4.41
Proprioceptive Shift Right	–3.7	12.8	1.24	2.55	1.32	3.94
Real questions Sync Left	–3	3	1.62	1.41	–1.21	1.05
Control questions Sync Left	–3	2.67	–0.85	1.59	0.3	–1.01
Real questions Async Left	–3	3	–1.01	1.71	0.43	–0.92
Control questions Async Left	–3	2.33	–1.47	1.51	0.91	–0.31
Real questions Sync Right	–2.67	3	1.35	1.54	–0.97	0.13
Control questions Sync Right	–3	3	–0.87	1.56	0.38	–0.64
Real questions Async Right	–3	3	–1.15	1.72	0.71	–0.62
Control questions Async Right	–3	2	–1.45	1.38	0.64	–0.49
Subjective Shift Left	–3.7	6	1.98	2.02	–0.05	–0.50
Subjective Shift Right	–2.7	6.7	1.9	1.97	0.4	–0.09
**PAI Clinical scales**						
Somatization (SOM)	37	84	50.9	10.1	1.11	1.12
Anxiety (ANX)	34	86	56.2	12.4	0.44	–0.40
Anxiety-Related Disorders (ARD)	35	81	56.1	10.0	0.10	–0.36
Depression (DEP)	35	91	52.1	11.3	1.20	1.44
Mania (MAN)	38	77	57.4	7.6	–0.14	–0.13
Paranoia (PAR)	35	79	51.3	9.2	0.73	0.10
Schizophrenia (SCZ)	33	87	55.8	11.5	0.52	0.01
Borderline Features (BOR)	42	89	59.6	9.5	0.69	0.42
Antisocial Features (ANT)	38	93	54.9	11.2	0.96	1.02
Alcohol Problems (ALC)	40	108	52.2	13.8	2.14	5.42
Drug Problems (DRG)	42	94	51.2	11.2	1.70	3.50
**Rorschach Domains**						
Engagement and Cognitive Processing	89.6	119.7	102.4	6.1	0.30	0.08
Perception and Thinking Problems	75.7	126.7	105.0	9.8	0.09	–0.10
Stress and Distress	87.6	139.9	106.2	11.6	0.77	0.31
Self and Other Representation	92.8	111.4	101.6	4.2	–0.06	–0.37

### Personality Assessment Inventory

Table 1 shows the descriptive statistics. Given that PAI scale scores are expressed in *T*-scores, in a non-clinical sample one would expect to obtain a mean value of 50T and a standard deviation of 10T. The mean values of the PAI clinical scales ranged from 50.9 T scores for the Somatization (SOM) scale to 59.6 *T* scores for the Borderline Features (BOR) scale. Overall, medium differences (i.e., Cohen’s *d* > 0.50) from the normative sample are observed for the Anxiety (ANX), Anxiety-Related Disorders (ARD), Mania (MAN), and Schizophrenia (SCZ) scales. The BOR scale mean value showed a large difference between our sample and the normative sample with the former obtaining a mean value of about 10 T scores greater than the mean value of the latter; however, this result is expected and is in line with the fact that age affects the BOR scale scores (Morey, 2007).

### Rorschach Performance Assessment System

Average summary scores for the R-PAS domains ranged from 101.6 to 106.2 SS (see Table 1). These values indicate that our participants’ scores were largely consistent with normative expectations. Indeed, the individual variables composing the domain scores reported in Table 1 are distributed as SS (which, by definition, have a mean of 100 and a standard deviation of 15). As such, none of the domain scores was more than half standard deviation apart from normative expectation (regarding this, it should be noted that in the field of personality assessment, “notable differences” are those consisting of a Cohen’s *d* greater than 0.50, i.e., greater than half a standard deviation). This result was expected, given that our participants were healthy volunteers.

### Correlations Between RHI and Personality Features

**FIGURE 2:**
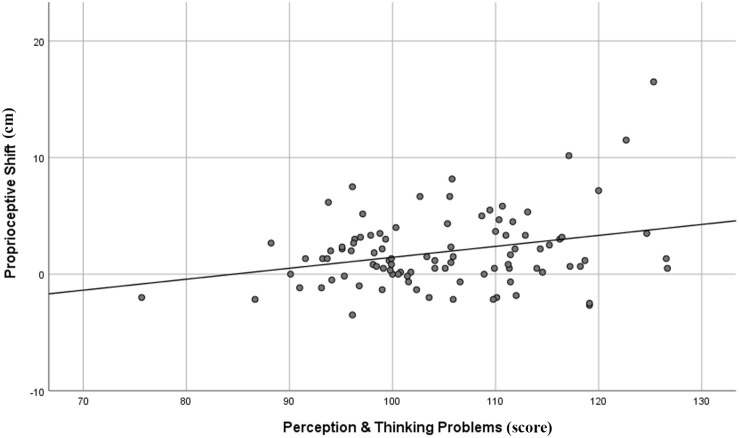
Graphical representation of the correlation between R-PAS Perception and Thinking Problems domain and proprioceptive shift of the left hand in the RHI paradigm.

**FIGURE 3:**
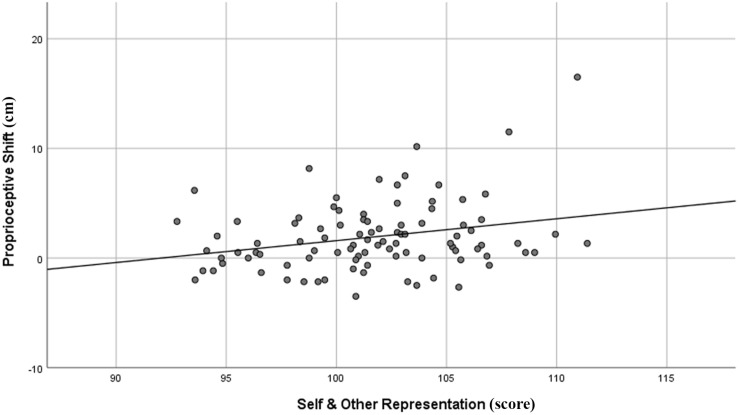
Graphical representation of the correlation between R-PAS Self and Other Representation domain and proprioceptive shift of the left hand in the RHI paradigm.

With respect to the PAI (see Table 2), neither the proprioceptive nor the subjective shift of both left and right hand correlated with any scales. Conversely, two Rorschach domains significantly correlated with the proprioceptive shift of the left hand: “Perception and Thinking Problems” (*r* = 0.28, 95% CIs = 0.08–0.45, *p* = 0.006) and “Self and Other Representation” (*r* = 0.26, 95% CIs = 0.06–0.44, *p* = 0.011) domains (see Table 3). In details, the higher was the proprioceptive shift, the higher was the score for both “Perception and Thinking Problems” (see Figure 2) and “Self and Other Representation” (see Figure 3). Applying the FDR procedure with α = 0.05 to all the correlations reported in Table 3 (i.e., both RHI indexes and both hands), “none of the them was significant”. However, at a less conservative alpha value of 0.10, the FDR procedure showed significant correlations between the “Perception and Thinking Problems” and “Self and Other Representation” domains and the proprioceptive shift of the left hand.

**TABLE 2:** Correlations between PAI Clinical scales and the RHI.

	**Proprioceptive Shift**						**Subjective Shift**					
		
	**Left hand**			**Right hand**			**Left hand**			**Right hand**		
				
	***R***	***95% CI***	***P***	***r***	***95% CI***	***p***	***r***	***95% CI***	***p***	***r***	***95% CI***	***p***
Somatization (SOM)	−0.07	−0.27 to 0.13	0.528	−0.11	−0.30 to 0.09	0.294	−0.14	−0.33 to 0.06	0.182	−0.04	−0.24 to 0.16	0.701
Anxiety (ANX)	0.00	−0.20 to 0.20	0.994	0.02	−0.18 to 0.22	0.809	−0.07	−0.27 to 0.13	0.497	0.02	−0.18 to 0.22	0.858
Anxiety-Related Disorders (ARD)	−0.04	−0.24 to 0.16	0.724	−0.09	−0.29 to 0.11	0.396	−0.14	−0.33 to 0.06	0.178	−0.04	−0.24 to 0.16	0.733
Depression (DEP)	0.10	−0.10 to 0.29	0.333	−0.12	−0.31 to 0.08	0.253	0.00	−0.20 to 0.20	0.978	−0.01	−0.21 to 0.19	0.910
Mania (MAN)	−0.01	−0.21 to 0.19	0.954	0.01	−0.19 to 0.21	0.903	0.06	−0.14 to 0.26	0.580	0.10	−0.10 to 0.29	0.310
Paranoia (PAR)	−0.05	−0.25 to 0.15	0.635	−0.19	−0.38 to 0.01	0.059	−0.08	−0.28 to 0.12	0.411	−0.03	−0.23 to 0.17	0.735
Schizophrenia (SCZ)	0.01	−0.19 to 0.21	0.923	−0.17	−0.36 to 0.03	0.095	−0.05	−0.25 to 0.15	0.599	0.00	−0.20 to 0.20	0.973
Borderline Features (BOR)	0.02	−0.18 to 0.22	0.821	0.06	−0.14 to 0.26	0.541	0.08	−0.12 to 0.28	0.410	0.13	−0.07 to 0.32	0.219
Antisocial Features (ANT)	0.01	−0.19 to 0.21	0.946	−0.02	−0.22 to 0.18	0.848	0.09	−0.11 to 0.29	0.368	−0.05	−0.25 to 0.15	0.607
Alcohol Problems (ALC)	0.09	−0.11 to 0.29	0.397	0.17	−0.03 to 0.36	0.099	0.00	−0.20 to 0.20	0.967	−0.05	−0.25 to 0.15	0.605
Drug Problems (DRG)	0.07	−0.13 to 0.27	0.498	−0.04	−0.24 to 0.16	0.700	0.00	−0.20 to 0.20	0.986	−0.07	−0.27 to 0.13	0.523

**TABLE 3:** Correlations between the R-PAS domains and the RHI.

	**Proprioceptive Shift**						**Subjective Shift**					
		
	**Left hand**			**Right hand**			**Left hand**			**Right hand**		
				
	***r***	***95% CI***	***p***	***r***	***95% CI***	***p***	***r***	***95% CI***	***p***	***r***	***95% CI***	***p***
Engagement and Cognitive Processing	−0.08	−0.28.12	0.453	−0.01	−0.21 to 0.19	0.926	0.06	−0.14 to 0.26	0.579	0.01	−0.19 to 0.21	0.887
Perception and Thinking Problems	0.28^∗^	0.08.45	0.006	0.00	−0.20 to 0.20	0.963	0.00	−0.20 to 0.20	0.973	−0.13	−0.32 to 0.07	0.210
Stress and Distress	−0.03	−0.23.17	0.768	−0.06	−0.26 to 0.14	0.562	−0.07	−0.27 to 0.13	0.492	−0.11	−0.30 to 0.09	0.304
Self and Other Representation	0.26^∗^	0.06.44	0.011	−0.09	−0.29 to 0.11	0.372	−0.07	−0.27 to 0.13	0.496	−0.01	−0.21 to 0.19	0.896

*^∗^Significant with False Discovery Rate (FDR) correction with α = 0.10.*

Additional analyses on the “Perception & Thinking Problems” domain (Table 4) showed that the proprioceptive shift in the left hand was positively correlated with Sum of Severe Cognitive Codes (SevCog; *r* = 0.21; 95% CIs = 0.01–0.39; *p* = 0.042) and Form Quality Minus Percent (FQ-%; *r* = 0.21; 95% CIs = 0.01–0.39; *p* = 0.039), and negatively correlated with Form Quality Ordinary Percent (FQo%; *r* = −0.30; 95% CIs = −0.47 to −0.11; *p* = 0.003). With respect to the “Self and Other Representation” domain (Table 5), the proprioceptive shift in the left hand was positively correlated with the Non-Pure Human to Sum Human Proportion (NPH/SumH; *r* = 0.29; 95% CIs = 0.13–0.44; *p* = 0.005), and negatively correlated with Whole Human content (H; *r* = −0.26; 95% CIs = −0.44, to −0.06; *p* = 0.012) and Cooperative Movement (COP; *r* = −0.20; 95% CIs = −0.39–0.00; *p* = 0.047). It should be noted, however, that only the correlation between the FQo% and the proprioceptive shift of the left hand remained significant after applying the FDR procedure with α = 0.05.

**TABLE 4:** Correlations between R-PAS Perception and Thinking Problems subcomponents and proprioceptive and subjective shifts of the RHI.

		**Proprioceptive Shift**		
		
**Perception and Thinking Domain**	**Psychological construct (Meyer et al., 2011)**	***r***	***95% CI***	***p***
**Page 1**				
Ego Impairment Index-3 EII-3	Thinking disturbance and severity of psychopathology	0.16	−0.04to0.35	0.126
Thought and Perception Composite TP-Comp	Problems in reality testing and thought disorganization	0.19	−0.01to0.38	0.059
Weighted Sum of Cognitive Codes WSumCog	Disturbed and disordered thought	0.07	−0.13to0.27	0.478
Sum of Severe Cognitive Codes SevCog	Psychotic-level disruptions in thought processes	0.21	0.01to0.39	0.042
Form Quality Minus percent FQ-%	Distortion or misinterpretation often leading to poor judgments or unconventional behavior	0.21	0.01to0.39	0.039
Percentage of W and D responses with FQ– WD–%	Distortion or misinterpretation occurring in perceptual situations that are more commonly selected	0.20	0.00to0.38	0.053
*Form Quality Ordinary percent* *FQo%*	Conventional judgment	–0.30^∗∗^	−0.47to−0.11	0.003
*Popular* *P*	Highly conventional and widely-accepted interpretations of the environment	–0.13	−0.32to0.08	0.216
**Page 2**				
Form Quality Unusual percent FQu%	Unconventional and individualistic ways of interpreting the world.	0.19	−0.01to0.38	0.063

*Descriptions of measured psychological constructs are taken almost literally from R-PAS manual (Meyer et al., 2011). ^∗∗^Significant with False Discovery Rate (FDR) correction with α = 0.05. Italics = reversed variables.*

**TABLE 5:** Correlations between R-PAS Self and Other Representation subcomponents and proprioceptive and subjective shifts of the RHI.

		**Proprioceptive Shift**		
		
**Self and Other Representation Domain**	**Psychological construct (Meyer et al., 2011)**	***r***	**95% CI**	***p***
**Page 1**				
Oral Dependency Language percent ODL%	Implicit dependent attitudes and behaviors	–0.04	−0.24to0.16	0.674
Space Reversal SR	Oppositionality and independence strivings	–0.04	−0.24to0.16	0.721
Mutuality of Autonomy-Pathology proportion MAP/MAHP	Poor object relations, including distorted schemas of self and other relationships	0.25^b^	0.05to0.43	0.339
Poor Human Representation proportion PHR/GPHR	Interpersonal competency and capacity for relatedness	0.19^b^	–0.01.38	0.072
Human Movement with FQ– M–	Atypical or distorted understanding of people that suggests disturbed interpersonal relations	0.04	−0.16to0.24	0.683
Aggressive content AGC	Aggressive concerns, preoccupations, and identifications	0.03	−0.17to0.23	0.759
Vigilance Composite V-Comp	Guardedness, effortful and focused cognition, sensitivity to cues of danger, and interpersonal wariness and distancing	–0.01	−0.21to0.19	0.940
*Human content* *H*	Ability to mentalize whole, intact humans	–0.26	−0.44to−0.06	0.012
*Cooperative Movement* *COP*	Propensity to view interactions as supportive, helpful, rewarding, and collaborative	–0.20	−0.39to0.00	0.047
*Mutuality of Autonomy-Health* *MAH*	Potential for mature and healthy interpersonal relationships	–0.18	−0.37to0.02	0.075
**Page 2**				
*All human content* *SumH*	Interpersonal interest and awareness.	–0.08	−0.28to0.12	0.456
Non-pure H proportion NPH/SumH	Tendency to view self and others in unrealistic or fanciful ways	0.29	0.10to0.46	0.005
Reflection r	Narcissistic-like or pleasurably self-involved traits	0.14	−0.06to0.33	0.161
Passive proportion p/(a + p)	Passive inclination or attitude (e.g., relying on others or surrendering to chance, luck, or fate)	0.00^b^	−0.20to0.20	0.991
Aggressive movement AGM	Aggressive intent or motive	–0.11	−0.30to0.09	0.293
Texture T	Interest in interpersonal closeness or contact	0.12^a^	−0.08to0.31	0.251
Personal knowledge justification PER	Tendency to justify one’s views and positions based on private, personal knowledge or authority	–0.16	−0.35to0.04	0.130
Anatomy An	Bodily, physical, or medical concerns	0.15	−0.05to0.34	0.136

*Descriptions of measured psychological constructs are taken almost literally from R-PAS manual (Meyer et al., 2011). ^*a*^Spearman correlation. Italics = reversed variables. ^*b*^Because both MAP/MAHP, PHR/GPHR, and p/(a+p) are proportions, R-PAS scoring program only calculates them if there are at least three relevant codes. For that reason, n decreases to 17 for MAP/MAHP, n decreases to 93 for PHR/GPHR, and n decreases to 95 for p/(a+p).*

## Discussion

In the present exploratory study, we aimed at investigating whether, how, and to what extent personality and psychopathological features were related to the RHI effect and, hence, could explain the strong inter-individual variability in its susceptibility. We thus analyzed the correlations between the two main behavioral indexes of the RHI and two personality assessment tools (i.e., the PAI and the R-PAS) in a relatively large group of healthy participants.

As regards the illusory effects and personality features, our sample showed results consistent with previous literature on normal populations. In the RHI, the participants displayed the ownership of the fake hand at both implicit (i.e., mislocalization toward the fake hand) and explicit (i.e., misattribution of the fake hand to the own body) levels, in line with other studies (Holmes and Spence, 2005; Tsakiris and Haggard, 2005; Costantini and Haggard, 2007; Petkova et al., 2011; Pyasik et al., 2018). Moreover, the higher mislocalization for the left than for the right hand replicates some previous data (Ocklenburg et al., 2011; Bertamini and O’Sullivan, 2014; Marotta et al., 2016; Dempsey-Jones and Kritikos, 2019). It is worth noting that neuropsychological studies showed that somatoparaphrenia (Vallar and Ronchi, 2009), anosognosia for hemiplegia (Piedimonte et al., 2016), hemianaesthesia (Pia et al., 2014), delusional body ownership (Pia et al., 2016), personal neglect (Halligan et al., 2003), supernumerary limbs (Halligan et al., 1993), autoscopic phenomena (Anzellotti et al., 2011), and others disease of bodily self-consciousness typically follow lesions of the right-hemisphere. Additionally, neuroimaging studies on intact brain functioning reported the key involvement of right sided premotor/parietal regions as well as right insular cortex in incorporating fake hands into the own body and in distinguishing it form external objects (Ehrsson et al., 2004, 2005; Tsakiris and Haggard, 2005; Tsakiris et al., 2008). Hence, the asymmetry of our findings might be explained because of the prominent role of the non-dominant hemisphere in body representation.

In the personality assessment tools, the average results of both the PAI and R-PAS in our sample were largely consistent with normative expectations (i.e., it is representative of the general, non-clinical, healthy adult population). With respect to the associations between the illusion and personality features, our results showed that the implicit index of the illusion (i.e., proprioceptive shift) of the left hand correlated to the implicit measure of stable personality features (i.e., the R-PAS). Specifically, the higher the mislocalization of the left hand in the synchronous respect to the asynchronous condition, the higher the scores in both “Perception and Thinking Problems” and “Self and Other Representation” domains. With respect to former domain, the proprioceptive shift of the left hand correlated positively with Form Quality Minus Percent (FQ-%) and Sum of Severe Cognitive Codes (SevCog), and negatively with Form Quality Ordinary Percent (FQo%). As for the second domain, the proprioceptive shift of the left hand correlated positively with Non-Pure Human to Sum Human Proportion (NPH/SumH) and negatively with Whole Human content (H) and Cooperative Movement (COP). However, the explicit measure of the illusion (i.e., subjective shift) was not associated with the explicit measure of personality and psychopathological features, namely the PAI. In the rest of the discussion, we will try to give an explanation of such correlations.

The first point to address regards the nature of the abovementioned association. The “Perception and Thinking Problems” domain addresses the tendency to not perceive/see things in a conventional manner (Meyer et al., 2011). In particular, the variable Form Quality is driven by the adherence of the response object to the shape of the blot as well as by the frequency at which it is identified. Indeed, the response process entails selecting the location along with retrieving object representations from memory and eventually leading to a final selection of a combination of the two in order to answer to the question “What might this be?”. Thus, subcomponent Form Quality ordinary (FQo) characterizes responses whose percepts are widely seen by others and are both conventional and accurate. In contrast, subcomponent Form Quality unusual (FQu) objects are less accurate (but not too inconsistent) with respect to the blot contours and are less frequent in the general population. In the end, subcomponent Form Quality minus (FQ−) responses are inaccurate, distorted, and infrequently seen (Meyer et al., 2011). In a whole protocol, a lower proportion of FQo (and, consequently, a higher proportion of FQu and/or FQ−) suggests that the subject imposes a personal and perhaps arbitrary contribution. For example, in Card V, the inkblot where most people see a butterfly or a bat, respondents with higher FQo score are likely to see those percepts, whereas those with lower FQo score are more likely to see different things (e.g., a human being, an airplane, a fire, and so on). Moreover, both FQo and FQ− are each significantly associated to psychotic disorders and other disorders with perceptual and cognitive disturbances, with a mean correlation of 0.48 and 0.49 respectively (Mihura et al., 2013). Although FQo% was the only Rorschach variable that showed a significant correlation with the Proprioceptive Shift after applying the FDR procedure with α = 0.05 and produced a 95% confidence interval that did not cross zero, from an interpretative point of view, FQo and FQ− could be considered the two opposite ends of the same construct, i.e., accurate *vs.* distorted perception. According to these considerations, the findings related to both FQo% and FQ−% might indicate that, on one end, the poorer the attitude to experience the world conventionally (i.e., low FQo% and high FQ−%), the higher the proprioceptive shift, and, on the other end, the higher the attitude to experience the world conventionally (i.e., high FQo% and low FQ−%), the lower the proprioceptive shift. Moreover, SevCog, which is an aggregate measure of the most severe disturbances of thought processes, showed a positive correlation (*p* = 0.042) with the proprioceptive shift. As is the case for the FQ−%, also the correlation between SevCog and RHI index did not remain significant after applying the FDR procedure and the 95% confidence interval ranged from 0.01 to 0.39. However, being another psychotic-related Rorschach subcomponent, SevCog may add strength to the interpretation of this domain, suggesting that also lapses of a psychotic-level in conceptualizing, reasoning, communicating, or organizing thought may be part of the illusion. In particular, SevCog captures a loosening of associations expressed at the level of visual images (e.g., seeing incongruent object combinations in the blot) and ideas (e.g., reporting a combination of concepts in contradictory/inappropriate ways). Indeed, in line with these hypotheses, the findings related to the “Perception and Thinking Problems” domain, allows us to hypothesize that a sort of proclivity to put together ideas and visual images in a more idiosyncratic way might be added to the tendency to perceive the world less accurately increasing the mislocalization of the own hand toward the fake hand. It is worth noting that the association between these psychosis-related processes in the Rorschach and the RHI is consistent with previous research that linked psychosis-proneness to RHI susceptibility (Asai et al., 2011; Thakkar et al., 2011; Germine et al., 2013; Kallai et al., 2015). Taken together, these data suggest that weaker body ownership, as indexed by the RHI, could be rooted in psychosis probe traits. Nonetheless, whereas the large majority of those studies found that using both implicit and explicit measures of the RHI, here the link is limited to the proprioceptive shift. The reason of this difference is still a left open question.

Interestingly, both FQ−% and SevCog, two measures often elevated in patients with psychotic disorders, produced weaker correlation values as compared with FQo%, which represents a measure of optimal, accurate perception. One may speculate that being a non-clinical sample, the absence of psychopathology might have reduced the amount of available data points for FQ−% and SevCog related analyses. As a result, a floor effect may have reduced the strength of the correlations between the proprioceptive shift and the Rorschach subcomponents related to psychopathology (i.e., FQ−% and SevCog), producing only small effect sizes. As such, recruiting both clinical and non-clinical participants would improve the strength of our correlation values.

With respect to “Self and Other Representation” domain, although none of the significant correlations remained significant after applying the FDR procedure at α = 0.05, two domain subcomponents, i.e., Non-Pure Human to Sum Human Proportion (NPH/SumH) and Whole Human content (H), produced 95% confidence intervals that did not cross zero (NPH/SumH: *r* = 0.29; 95% CIs = 0.10–0.46; *p* = 0.005; H: *r* = −0.26; 95% CIs = −0.44 to −0.06; *p* = 0.012). Similarly to FQo and FQ−, these two subcomponents are inversely related to each other, in that H is part of SumH, the denominator of NPH/SumH. Indeed, these subcomponents refer to the R-PAS coding category “content,” which taps the representations that are present in the respondent’s mind when answering the question “What might this be?” (Meyer et al., 2011). While H represents the number of whole, intact humans seen in the inkblots, the NPH/SumH refers to the proportion of unreal or partial human figures (e.g., a witch, a devil, the nose of a person, a leg of a human being, and so on) compared to the total number of humans seen in the inkblots. Said differently, both subcomponents represent the way the test-taker sees human beings in the inkblots, differentiating between realistic, complete figures (high H and low NPH/SumH) and unrealistic or partial figures (low H and high NPH/SumH). These two subcomponents evaluate one’s own ability to perceive and experience the human beings in a realistic and integrated (rather than unrealistic and/or partial) manner. Indeed, elevations of NPH/SumH reflect a tendency to represent, perceive, and experience others in unrealistic and fanciful ways, or to “mentally playout interpersonal scenarios in such way” (Meyer et al., 2011). As a result, these findings seem to suggest that the tendency to see one’s own or other’s bodies in an unrealistic way (i.e., high scores on NPH/SumH and low scores on H) could further increases the mislocalization of the own hand toward the fake hand. However, given the larger number of comparisons, these results could express a not true effect. Hence, whether a tendency to represent the body in unrealistic/fanciful ways increases the illusory effects is still something to be addressed in future studies.

Overall, our results seem to indicate that persistent personality features, despite not being directly related to sensory perception, may actually influence the illusory effects, which are indeed related to specific types of sensory stimulation (i.e., visual, tactile, and proprioceptive). This is not trivial but, rather, consistent with evidence showing that, besides multisensory integration (i.e., the main process subserving the RHI), additional factors might influence the RHI (Durgin et al., 2007; Asai et al., 2011; Thakkar et al., 2011; Tsakiris et al., 2011; Eshkevari et al., 2012; Haans et al., 2012; Germine et al., 2013; Kallai et al., 2015; Costantini et al., 2016; Marotta et al., 2016). Here we suggest that personality might strongly contribute to explain the inter-individual variability of the illusion. In details, perceiving unconventionally, binding thoughts/images idiosyncratically (and perhaps also seeing unrealistic bodies) may increase the implicit measure of the RHI (i.e., the mislocalization of the own hand toward the fake hand) in a top-down manner. Broadly speaking, this could be considered at least reasonable, given that the RHI is essentially perceiving a coherent but impossible percept. A key component for the occurrence of this illusory experience is shifting the felt position of the own hand toward the fake hand. Perhaps, this might explain why experiencing alterations in perception and behavior (i.e., suggestibility) is linked to higher illusory effects (Walsh et al., 2015; Marotta et al., 2016). Specifically speaking, it is worth noting that some previous studies claimed a tight link between an altered experience of the own body as well as of its boundaries and multisensory integration processes (Nelson et al., 2009; Gallese and Ferri, 2014). The idea is rooted in developmental literature showing that the coherence of bodily self and the ability to merge incoming sensory signals (particularly when useful for the self-other distinction) go hand in hand (Rochat and Striano, 2002). Accordingly, we can speculate that people with issues in “Perception and Thinking Problems” and (to some extent) “Self and Other Representation” might alter perceptual integration as well as the boundaries of the bodily self, which means an increased probability to perceive an external object as part of one’s own body.

The second point to address refers to the specificity of the association between personality traits and illusory effects. As already mentioned, “Perception and Thinking Problems” and “Self and Other Representation” were correlated with the implicit (but not to the explicit) measures of the RHI and with the left, but not with the right, hand. With respect to the former point, the same association with mislocalization only it has been already reported by a previously mentioned study with the suggestibility (Walsh et al., 2015). A possible speculative interpretation of this datum might be that the R-PAS is a performance-based assessment tool. In other words, scores are obtained by means of implicit responses, exactly as it happens with the mislocalization toward the rubber hand that does not measure directly the experience of owning the fake hand. As regards the latter point, the possible interpretation might be the hemispheric lateralization. Indeed, Hiraishi et al. (2012) showed that the Rorschach task is associated with the non-dominant hemisphere (which controls the left hand in right-handed subjects). The common variability shared between the Rorschach task and the RHI on the left hand conceived in terms of hemispheric laterality could thus shed light on the observed correlation between R-PAS variables and the RHI experience assessed on the left hand. Alternatively (or, perhaps, in addition), another factor that is likely to have contributed to the observed left-hand correlations is that, in our sample, the proprioceptive shift was stronger for the left hand rather than for the right. Hence, since correlation is a covariation, if one variable has low variability, its covariation with other variables also will be flat. Said differently, it is possible that a floor effect occurred, meaning that the proprioceptive shift on the right hand was too small and lacked the variability needed to give rise to an appreciable covariance with the R-PAS variables.

Although this study has evaluated systematically the association between the RHI and personality features assessed by both self-report and performance-based instruments, some limits are worth mentioning. Firstly, the absence of significant correlations between the explicit measure of personality and the explicit measure of RHI may rely on the clinical nature of the PAI, a tool developed to capture psychopathological features rather than healthy personality functioning. As such, participants in total did not show any evident psychopathological features reducing the variability of the PAI clinical scale scores. Secondly, any correlation reveals whether a relationship between variables exists but cannot prove that changes to one variable lead to changes to another variable. Hence, future studies should be intended to prove causal relationships. This, in turn, would crucially help to clarify how and to which extent personality has a role in the emergence of the RHI. Thirdly, with our limited power, among the Rorschach domains the two correlations with *p* < 0.05 remained significant after applying the FDR procedure only at a less conservative alpha value of 0.10 (i.e., Perception and Thinking Problems and Self and Other Representation), whereas among all the correlations computed for the subcomponents, only one remained significant after applying the FDR procedure at α = 0.05 (i.e., between the Proprioceptive Shift for the left hand and FQo%). Future studies may evaluate the strength of these relations. Lastly, our sample was largely imbalanced in terms of gender. Because only 19 out of 96 participants were men, we could not compute additional analyses to evaluate the impact of gender.

To conclude, our exploratory study may have shed some light on the possible role played by personality features in the RHI effects. The most compelling result refers to the relationship between the tendency to perceive unconventionally and mislocalizing the own hand toward the fake hand. While this correlation was significant even in our sample of non-clinical individuals, the degree of the contribution of putting together ideas/visual images in an idiosyncratic way and seeing unrealistic bodies remain uncertains. As a more general, concluding remark, the results obtained in this exploratory study indicate that future research should focus on those personality characteristics that concerns (meta)cognitive processes, executive functioning, thought organization and reality testing, schizotypy, psychosis, and/or fantasy proneness in both clinical and non-clinical samples.

## Data Availability Statement

The datasets generated for this study will not be made publicly available. Requests to access the datasets should be directed to the corresponding author.

## Ethics Statement

The studies involving human participants were reviewed and approved by the Bioethical Committee of University of Turin. The patients/participants provided their written informed consent to participate in this study.

## Author Contributions

DB, LP, and LG designed the study. FA and DG collected the data for personality tests. MA and LC collected the data for RHI. MP and CP ran the statistical analyses. All the authors contributed to data interpretation and manuscript preparation.

## Conflict of Interest

The authors declare that the research was conducted in the absence of any commercial or financial relationships that could be construed as a potential conflict of interest.

## Footnotes

1 R-PAS administrations typically last about 30–45 min, but there might be some extra-variability from one case to another (Meyer et al., 2011).
