# Supplementary material for: Relationships Between Personality Features and the Rubber Hand Illusion: An Exploratory Study
Source: Front Psychol. 2019 Dec 10;10:2762. doi: 10.3389/fpsyg.2019.02762 (PMC6914866; doi:10.3389/fpsyg.2019.02762)
Supplement: Supplementary file 1 [file Data_Sheet_1.DOCX]

**RHI Questionnaire**

Q1. It felt as if I was feeling the stroking touch in the location where I saw the rubber hand touched

Q2. It seemed as though the touch I felt was caused by the paintbrush touching the rubber hand.

Q3. I felt as if the rubber hand was my hand

Q4. It felt as if my hand was drifting towards the left/right (towards the rubber hand)

Q5. It seemed as if the touch I was feeling came from somewhere between my own hand and the rubber hand

Q6. It felt as if my hand was turning ‘rubbery’

**The Personality Assessment Inventory (PAI) Clinical Scales**

Somatization (SOM)

Anxiety (ANX)

Anxiety-Related Disorders (ARD)

Depression (DEP)

Mania (MAN)

Paranoia (PAR)

Schizophrenia (SCZ)

Borderline Features (BOR)

Antisocial Features (ANT)

Alcohol Problems (ALC)

Drug Problems (DRG)

**Rorschach Performance Assessment System domains**

Engagement & Cognitive Processing

Perception & Thinking Problems

Stress & Distress

Self & Other Representation
